# Bispecific Engineered Antibody Domains (Nanoantibodies) That Interact Noncompetitively with an HIV-1 Neutralizing Epitope and FcRn

**DOI:** 10.1371/journal.pone.0042288

**Published:** 2012-08-07

**Authors:** Rui Gong, Yanping Wang, Tianlei Ying, Dimiter S. Dimitrov

**Affiliations:** 1 Protein Interactions Group, Frederick National Laboratory for Cancer Research, National Institutes of Health, Frederick, Maryland, United States of America; 2 SAIC-Frederick, Inc., Frederick, Maryland, United States of America; National Cancer Institute, NIH, United States of America

## Abstract

Libraries based on an isolated human immunoglobulin G1 (IgG1) constant domain 2 (CH2) have been previously diversified by random mutagenesis. However, native isolated CH2 is not very stable and the generation of many mutations could lead to an increase in immunogenicity. Recently, we demonstrated that engineering an additional disulfide bond and removing seven N-terminal residues results in an engineered antibody domain (eAd) (m01s) with highly increased stability and enhanced binding to human neonatal Fc receptor (FcRn) (Gong et al, JBC, 2009 and 2011). We and others have also previously shown that grafting of the heavy chain complementarity region 3 (CDR-H3 (H3)) onto cognate positions of the variable domain leads to highly diversified libraries from which a number of binders to various antigens have been selected. However, grafting of H3s to non-cognate positions in constant domains results in additional residues at the junctions of H3s and the CH2 framework. Here we describe a new method based on multi-step PCR that allows the precise replacement of loop FG (no changes in its flanking sequences) by human H3s from another library. Using this method and limited mutagenesis of loops BC and DE we generated an eAd phage-displayed library. Panning of this library against an HIV-1 gp41 MPER peptide resulted in selection of a binder, m2a1, which neutralized HIV-1 isolates from different clades with modest activity and retained the m01s capability of binding to FcRn. This result provides a proof of concept that CH2-based antigen binders that also mimic to certain extent other functions of full-size antibodies (binding to FcRn) can be generated; we have previously hypothesized that such binders can be made and coined the term nanoantibodies (nAbs). Further studies in animal models and in humans will show how useful nAbs could be as therapeutics and diagnostics.

## Introduction

Engineered monoclonal antibodies (mAbs) based on immunoglobulins (Igs) have been highly successful for therapy of various diseases [1]–[6]. However, full-size mAbs may not efficiently penetrate tissues (e.g. solid tumors) and/or bind to regions on the surface of some molecules (e.g., the HIV envelope glycoprotein (Env)) that are accessible by molecules of smaller size [7]. Several protein scaffolds based on Ig and non-Ig domains have been developed to overcome these limitations [8]–[15]. A major drawback of most of those scaffolds and corresponding binders is that they lack full-size mAb functions conferred by the Ig Fc that can bind to Fc receptors including the neonatal Fc receptor (FcRn) which is important for enhanced half-life *in vivo* [16].

We have proposed that human Ig constant CH2 domain (CH2 of IgG, IgA and IgD, and CH3 of IgE and IgM) could be used as a novel scaffold because it contains binding sites or portion of binding sites conferring stability and effector functions, which might offer additional advantages compared to other scaffolds [7]. For such CH2-based antigen binders we coined the term “nanoantibodies (nAbs)” [7].

In a previous study, we selected a binder against the Env gp120 from a library based on wild-type CH2 (wtCH2) scaffold where the residues in two loops (BC and FG) were mutated to four residues (Y, A, D, or S) [17]. However, the wtCH2 domain has significantly lower thermal stability compared to other small scaffolds such as the tenth type III domain of human fibronectin [18]–[20]. Furthermore, the pH-dependent binding of wtCH2 to a single chain soluble recombinant human FcRn (shFcRn) [21] was very weak if any [22]. In order to increase the stability, we identified a CH2 variant, m01s, by engineering an additional disulfide bond and removal of seven unstructured residues at the N-terminus, which exhibits not only significantly increased thermal stability but also enhanced binding to shFcRn [19], [22]. We have hypothesized that binders selected from m01s-based libraries could bind to antigens and retain binding to FcRn.

**Table 1:** Primers for precisely grafting VH H3 onto the m01s loop FG.

Step	Primer	
I	Upstream primer: VHH3m01sLoopFG IF	5′ TAC GCC DYR TAT TAC TGT 3′
	Downstream primer: VHH3m01sLoopFG IR	5′ GGT GGT GCC CTG GCC CCA 3′
II	Upstream primer: VHH3m01sLoopFG IIF	5′ TAC AAG DYR TAT TAC TGT 3′
	Downstream primer: VHH3m01sLoopFG IIR	5′ GGT GCA GCC CTG GCC CCA 3′
III	Upstream primer: VHH3m01sLoopFG IIIF	5′ TAC AAG TGC TAT TAC TGT 3′
	Downstream primer: VHH3m01sLoopFG IIIR	5′ GGT GCA CTC CTG GCC CCA 3′
IV	Upstream primer: VHH3m01sLoopFG IVF	5′ TAC AAG TGC AAG TAC TGT 3′
	Downstream primer: VHH3m01sLoopFG IVR	5′ GGT GCA CTC GAT GCC CCA 3′
V	Upstream primer: VHH3m01sLoopFG VF	5′ TAC AAG TGC AAG GTC TGT 3′
	Downstream primer: VHH3m01sLoopFG VR	5′ GGT GCA CTC GAT GGG CCA 3′
VI	Upstream primer: VHH3m01sLoopFG VIF	5′ TAC AAG TGC AAG GTC AGT 3′
	Downstream primer: VHH3m01sLoopFG VR	5′ GGT GCA CTC GAT GGG CCA 3′

* D  =  A + T + G, Y  =  C + T, R  =  A + G.

We have previously showed that grafting of the light chain variable domain (VL) complementarity region 3 (CDR-L3 (L3)) to heavy chain variable domain (VH) CDR1 (CDR-H1 (H1)) could further increase the diversity and therefore give more opportunities for selection of binders [23]. Comparing the structure of CH2 with that of a VH binder against HIV-1 gp120 (m36) [24], we found that loops BC and FG in CH2 could be appropriate for grafting by H1 and VH CDR3 (CDR-H3 (H3)), respectively. Therefore, we developed a new method based on multi-step PCR to precisely graft H3 onto loop FG without changing the amino acids in the framework. We also mutagenized loops BC and DE. This library with grafted H3s onto FG and mutagenized loops BC and DE was used for selection of an HIV-1 neutralizer m2a1 targeting a peptide sp62 from the HIV-1 Env membrane proximal external region (MPER). Importantly, m2a1 retained the capability of the scaffold m01s to bind to shFcRn in a pH-dependent manner, which might extend its half-life *in vivo*. To our knowledge m2a1 is the first reported eAd that can bind simultaneously to an antigen (sp62) and to an Fc receptor (FcRn), i.e., it is what we termed nanoantibodies (nAbs).

**Table 2:** Primers for rational mutagenesis of the m01s loop BC.

Position	Change to	Codon*	Comment	Diversity
D	D and G	GRT	–	2
V	V, F and Y	KWT	An unexpected “D” will be introduced	4
S	neutral hydrophilic amino acid: N, T and S	WMC	An unexpected “Y” will be introduced	4
H	any amino acid	NNY	No “K, Q, E, W, M”	15
E	any amino acid	NNY	No “K, Q, E, W, M”	15
D	Deleted	–	–	
P	non-acidic amino acid: P, Y and S	YMT	An unexpected “H” will be introduced	4
E	mainly hydrophobic amino acid	NHT	Unexpected several hydrophobic amino acids will be introdued	12
V	limited hydrophobic amino acid: A, V, Y and F	KHT	Unexpected “D” and “S” will be introduced	6

* R  =  A + G, K  =  T + G, W  =  A + T, M  =  A + C, N  =  A + C + G + T, Y  =  C + T, H  =  A + T + C.

## Materials and Methods

### Cells, Viruses, Peptides and Antibodies

We purchased the 293 T cells from ATCC. Other cell lines and plasmids used for expression of various HIV-1 Envs were obtained from the National Institutes of Health AIDS Research and Reference Reagent Program (ARRRP). The biotin-sp62 peptide (QQEKNEQELLELDKWASLWN) from the HIV-1 Env MPER and biotin-scrambled sp62 peptide were commercially synthesized by CPC Scientific (CA).

**Table 3:** Primers for limited mutagenesis of the m01s loop DE.

Position	Change to	Codon*	Comment	Diversity
Y	hydrophobic amino acid	KHT	Unexpected “D” and “S” will be introduced	6
N	neutral hydrophilic amino acid	WMC	An unexpected “Y” will be introduced	4
S	neutral hydrophilic amino acid	WMC	An unexpected “Y” will be introduced	4
T	neutral hydrophilic amino acid	WMC	An unexpected “Y” will be introduced	4
Y	hydrophobic amino acid	KHT	Unexpected “D” and “S” will be introduced	6

* K  =  T + G, H  =  A + T + C, W  =  A + T, M  =  A + C.

### Construction of m01s with Grafted H3s

To test if H3 could be grated onto loops in m01s, three constructs (m01sLBCm36H3: replacement of m01s loop BC by m36 H3; m01sLFGm36H3: replacement of m01s loop FG by m36 H3; m01sLFGVHH3: replacement of m01s loop FG by an H3 randomly selected from our VH library [25]) were made based on phagemid pComb3X. These clones were verified by direct sequencing and used for transformation of the *E. coli* HB2151 for expression and purification as described previously [19].

**Figure 1:**
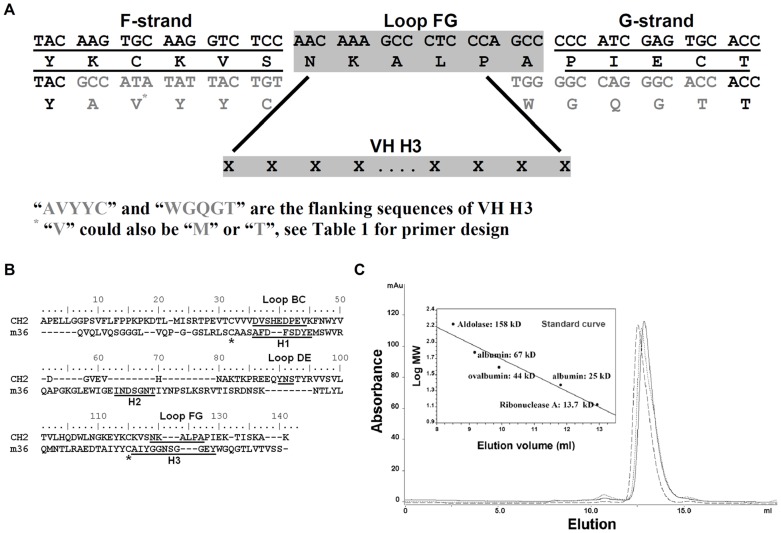
Grafting of VH H3 onto m01s loop FG. (A) A schematic for grafting VH H3 to m01s loop FG. (B) Alignment of CH2 with m36. (C) Size exclusion chromatography of m01sLBCm36H3 (–), m01sLFGm36H3 (–) and m01sLFGVHH3 (⋅⋅⋅). The insert is a standard curve.

### Oligomer Formation of m01s after Grafting

Estimation of oligomer formation of the purified m01sLBCm36H3, m01sLFGm36H3 and m01sFGVHH3 was performed by size exclusion chromatography (SEC) (Superdex 75 10/300 GL, GE healthcare, UK). A gel-filtration of standards consisting of Aldolase (158 kD), Bovine serum albumin (67 kD), Ovalbumin (44 kDa), Chymotrypsinogen A (25 kD) and Ribonuclease A (13.7 kDa) was used to define the molecular weight.

**Figure 2:**
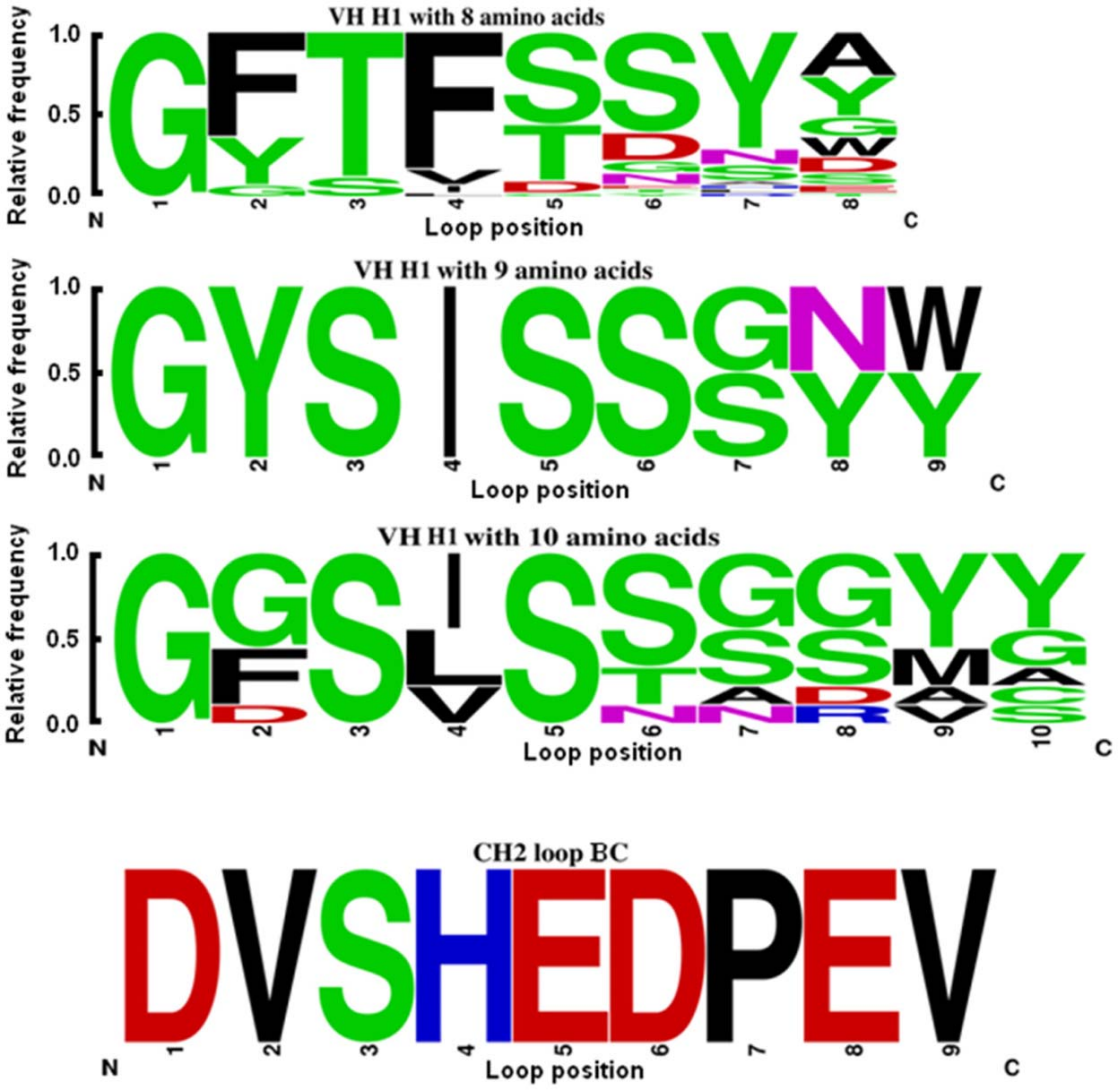
Comparison of CH2 loop BC and germline VH H1. The frequency of amino acids occurring in H1 according to IMGT website was calculated by WebLogo. The amino acids were colored as follows: polar G, S, T, Y, C (Y is classified as “polar” amino acids by WebLogo and excluded from the “hydrophobic” amino acids listed below although it is also hydrophobic): green; neutral Q, N: purple; basic K, R, H: blue; acidic D, E: red; hydrophobic A, V, L, I, P, W, F, M: black. The dominant length of H1 is eight amino acids (41 from 53 analyzed sequences); three sequences are nine amino acid long and nine sequences are ten amino acid long.

### Library Construction

A newly developed method based on “multi-step PCR” was used to precisely graft H3 from our previously constructed highly diversified human VH-based libraries [25] to m01s loop FG without changing the flanking sequences. Briefly, only one amino acid was changed by using one pair of primers at each PCR step. For example, in the first step, primer VHH3m01sLoopFG IF and VHH3m01sLoopFG IR (Table 1) were used to amplify H3s form VH library. In the second step, primer VHH3m01sLoopFG IIF and VHH3m01sLoopFG IIR were used to amplify the H3s from I round. In this step, two amino acids “A” and “T” in the flanking sequences of H3 were changed to the corresponding amino acids “K” and “C” in m01s strand F and G, respectively. According to this method, after VI step PCR, all the amino acids in the flanking sequences of H3 were replaced by the corresponding amino acids in m01s F and G strands, while only H3 was inserted onto loop FG.

**Figure 3:**
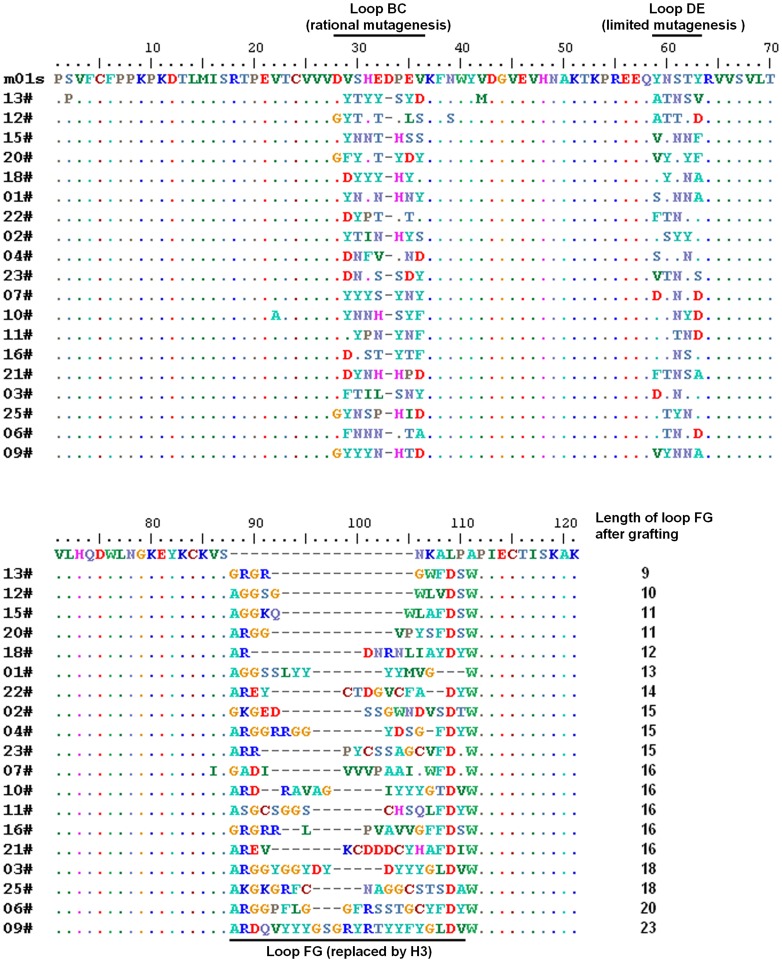
Sequence alignment of randomly selected clones with 8 amino acid mutations in loop BC, 5 amino acid mutations in loop DE, and VH H3 grafted onto loop FG in the m01s-based library. Nineteen of twenty five clones (76%) were expressed correctly and are shown here.

Degenerate primers (Table 2) were used to introduce mutations in m01s loop BC according to the comparison of H1 germline sequence in IMGT data base [26]–[33] analyzed by WebLogo [34], [35]. For example, in some positions like 2 and 3 in H1, the dominant amino acids were hydrophobic and neutral, respectively, while the amino acids in the same positions of m01s loop BC were also hydrophobic (valine) and neutral (serine), respectively. Then, these amino acids were mutated to any hydrophobic and neutral amino acids, respectively. In other positions, if there was no consistence, the amino acids were mutated according to the occurrence of the corresponding partners in H1. We also found the m01s with eight amino acids in loop BC showed better soluble expression level than that with nine and ten amino acids (data not shown). Therefore, we deleted one amino acid in the tip of loop BC and designed primers to rationally mutate the rest eight amino acids.

**Figure 4:**
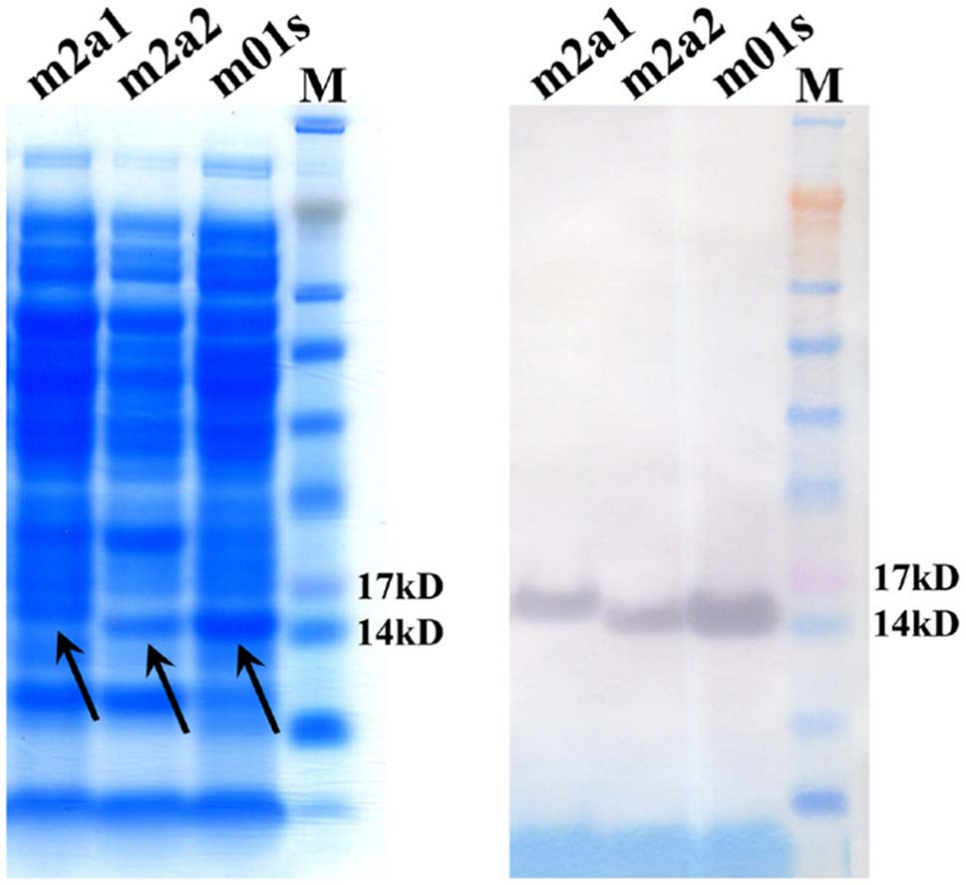
Soluble expression of m2a1 and m2a2 after panning against sp62 as tested by SDS-PAGE (left) and Western blot (right). Soluble expression of m2a1 and m2a2 is indicated by arrows.

Because m01s loop DE could not match VH CDR2 (CDR-H2 (H2)), we mutated the original amino acid without changing its property (Table 3). For example, “Y”, a hydrophobic amino acid in loop DE, was changed to any other hydrophobic amino acids for the library construction. “T” and “Y” were also mutated to further increase the diversity.

**Figure 5:**
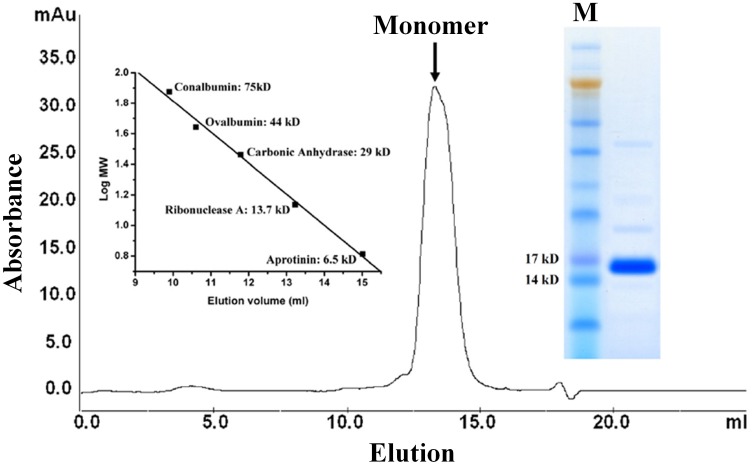
Size exclusion chromatography of purified m2a1. The left insert is a standard curve while the right one shows purified m2a1. Calculated M.W. according to amino acid sequence of m2a1 is 15.4 kD.

Intact PCR fragments were amplified from splicing by overlap extension (SOE) PCR and subjected to SfiI digestion and ligated to the pComb3X. The ligated product was desalted and transformed to the electrocompetent TG1 cells using an electroporator (Bio-Rad, CA) to make the phage-displayed library according to published protocols [36].

**Figure 6:**
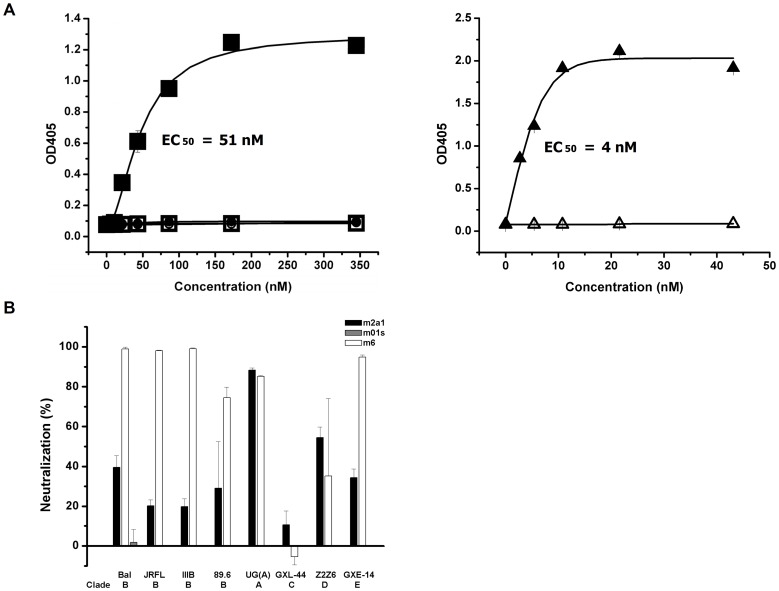
Binding of m2a1 to sp62 and neutralization of HIV-1. (A) Left: binding of m2a1 (▪) (EC_50_ = 51 nM) and m01s (•) to sp62; binding of scrambled sp62 to m2a1 (□), m01s (○) was used as negative control. Right: binding of m66.6 to sp62 (▴) and scrambled sp62 (Δ). (B) Percentage inhibition of a panel of viruses pseudotyped with Envs of HIV-1 primary isolates by m2a1 at 5 µM (77 µg/ml) and m6 (positive control) at 0.5 µM (14 µg/ml), respectively. m01s at 5 µM (70 µg/ml) was used as negative control. Data are presented as mean ± SD.

### Selection, Expression and Purification of Binders

A biotin-sp62 peptide was used as antigen for selection of binders from m01s-based library with magnetic beads (Dynabeads® MyOne™ Streptavidin T1, Invitrogen, CA). After five rounds of panning, ten clones were sent for DNA sequencing. From 10 clones, 9 had correct open reading frame. Two dominant clones m2a1 (three copies) and m2a2 (four copies) were transferred into HB2151 for expression which was checked by SDS-PAGE and Western-blot. In Western-blot, the mouse anti-His tag monoclonal antibody (Applied Biological Materials Inc., Canada) and alkaline phosphatase (AP)-goat-anti-mouse IgG polyclonal antibody (Sigma-Aldrich, MO) were used as primary and secondary antibodies, respectively. Both m2a1 and m2a2 were purified by the same method described previously [19]. However, the purification of m2a2 was difficult due partially to some aggregation, and needed to be further optimized. Only m2a1 was chosen for further characterization.

**Figure 7:**
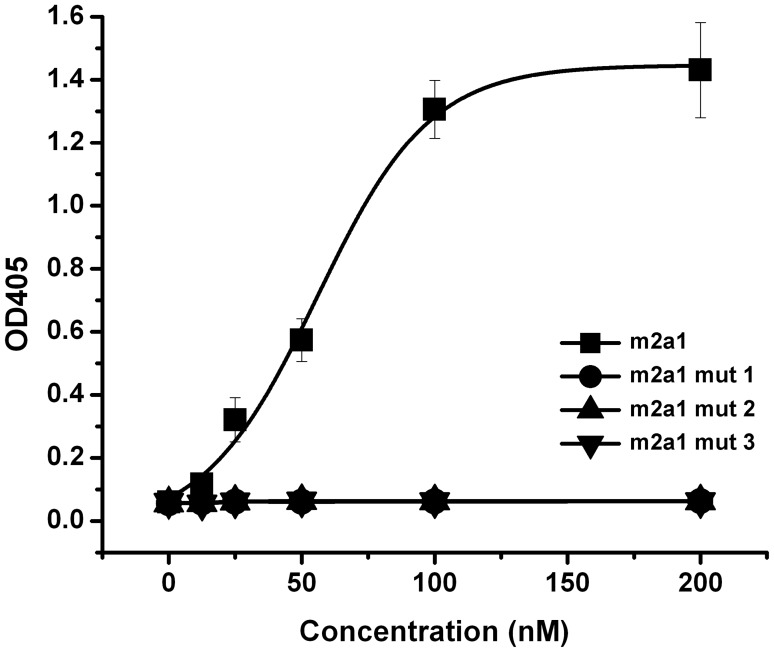
Binding of m2a1 and mutants to sp62 (mut 1– replaced loop BC in m2a1 to its original sequence in m01s; mut 2– replaced loop DE in m2a1 to its original sequence in m01s; mut 3– replaced loop FG in m2a1 to its original sequence in m01s).

### Estimation of Molecular Weight of m2a1 in Solution

Estimation of molecular weight of the purified m2a1 was performed by SEC as described above. A gel-filtration of standards consisting of Conalbumin (75 kD), Ovalbumin (44 kD), Carbonic Anhydrase (29 kD), Ribonuclease A (13.7 kD) and Aprotinin (6.5 KD) was used to define the molecular weight.

**Figure 8:**
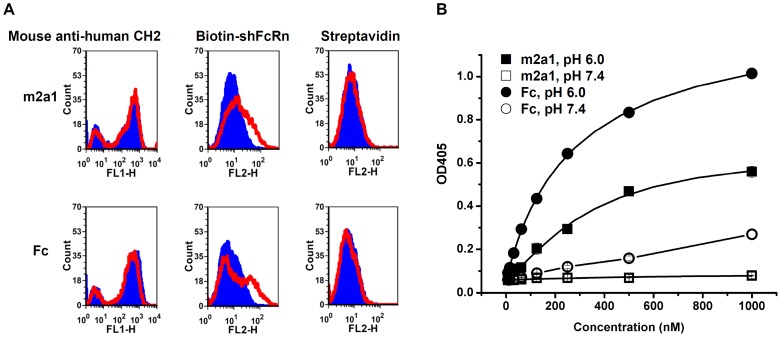
Binding of m2a1 to shFcRn. (A) Binding of yeast-expressed m2a1 and Fc to shFcRn at pH 6.0 (red) and pH 7.4 (blue) measured by flow cytometry. Fluorescence intensity shifts were observed in both cases of m2a1 and Fc. The expression of m2a1 and Fc on yeast cell surface was detected by the mouse anti-human CH2 mAb. PE-streptavidin was used as negative control. (B) Binding of m2a1 and Fc to shFcRn tested by ELISA. The binding of m2a1 to shFcRn at pH 6.0 (▪) and pH 7.4 (□) was tested while that of Fc to shFcRn at pH 6.0 (•) and pH 7.4 (○) was used as control. m2a1 showed good pH-dependent binding to shFcRn although the EC_50_ at pH 6.0 was lower than that of Fc to shFcRn. Data presented as mean ± SD.

### Binding of m2a1 to sp62 Assay

ELISA was used for measurement of the binding. Purified m2a1 (8 µg/ml) was coated to the 96-well plate at 4°C overnight. m66.6 (8 µg/ml), a previously identified HIV-1 broadly neutralizing mAb targeting MPER [37] and m01s (8 µg/ml) were used as positive and negative controls, respectively. The biotin-sp62 peptide with serial dilutions was pre-mixed with Streptavidin-Peroxidase Polymer (Sigma-Aldrich, MO) before adding into the plate. Biotin-scrambled sp62 was used as negative controls. ABTS (Roche, IN) was used to develop color and OD405 was taken 5–10 min afterward. All of the ELISA experiments were performed in duplicate in this study.

**Figure 9:**
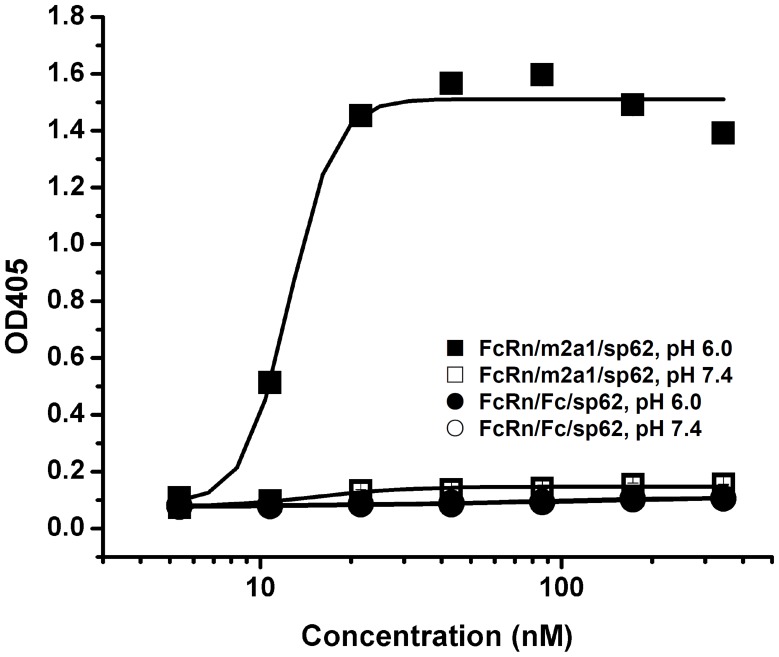
Binding of m2a1 to sp62 in the presence of shFcRn. The binding of shFcRn-bound m2a1 to sp62 was measured at pH 6.0 (▪) and pH 7.4 (□) by ELISA. m2a1 could still bind to sp62 after binding to shFcRn at pH 6.0. Binding of shFcRn-bound Fc to sp62 at pH 6.0 (•) and pH 7.4 (○) was used as control. Data presented as mean ± SD.

### Neutralization Activity of m2a1 Assay

The cell line-based assay was carried out in HOS CD4+CCR5+target cells containing a tat-inducible luciferase reporter that express CD4 and CCR5. Infectivity titers were determined on the basis of luminescence measurements at 3 days post-infection of the cells by pseudotyped viruses. Neutralization assays were carried out in triplicate wells by pre-incubation of m2a1 (5 µM, 77 µg/ml) with pseudotype viruses for 30 min at 37°C followed by infection of 1–2×10^4^ HOS CD4+CCR5+cells. The degree of virus neutralization by antibody was achieved by measuring luciferase activity. Luminescence was measured after 3 days. The mean luminescence readings for triplicate wells were determined. A single-chain variable fragment (scFv) m6 (0.5 µM, 14 µg/ml) [38] against HIV-1 Env and m01s (5 µM, 70 µg/ml) was used as positive and negative controls, respectively.

**Figure 10:**
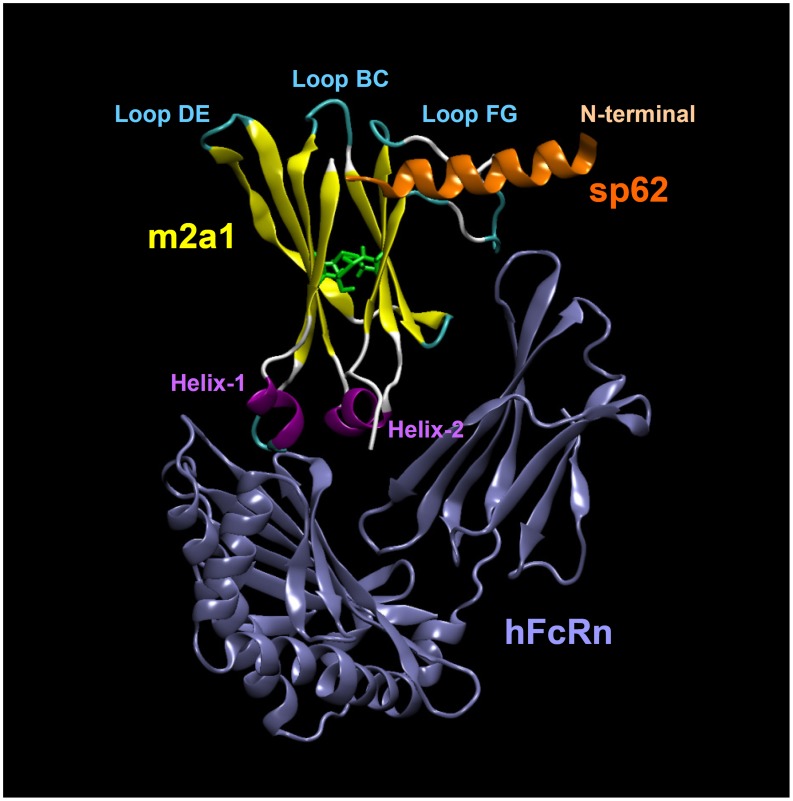
Molecular modeling and docking of the hFcRn/m2a1/sp62 complex. As predicted by modeling and docking, m2a1 interacts with sp62 by the loop FG, which is similar to the binding of 2F5 to sp62; and bind to FcRn by the residues around helix-1, which is consistent with the currently known FcRn binding site on the CH2 domain of Fc.

### Binding of m2a1 Mutants to sp62

In order to investigate whether all of the three loops are involved in binding to sp62, three mutants: m2a1 mut 1 (replacement of loop BC in m2a1 to its original sequence in m01s), m2a1 mut 2 (replacement of loop DE in m2a1 to its original sequence in m01s) and m2a1 mut 3 (replacement of loop FG in m2a1 to its original sequence in m01s) were constructed. Same methods described above were used for expression and purification of these mutants, and analysis of the binding of these mutants to sp62.

### Construction of m2a1 for Yeast Surface Expression

m2a1 was cloned into pYD7 vector which was a modified version from pCTCON2 [39] with moving the agglutinin protein aga2p to the C-terminal of interest proteins as construction of pYD7-Fc in our previous work [22]. The correct clone was verified by direct sequencing. The construct was transformed into EBY100 cells for surface expression according to the protocol described previously [39].

### Binding of m2a1 to shFcRn Measured by Flow Cytometry

For measurement of the binding of m2a1 to purified shFcRn [21] (produced in our lab), yeast cells containing pYD7-m2a1 were grown in SDCAA medium (20 g/liter dextrose, 6.7 g/liter Difco yeast nitrogen base w/o amino acid, 5 g/liter Bacto casamino acids, 5.4 g/liter Na_2_HPO_4_, and 8.56 g/liter NaH_2_PO_4_ H_2_O) and then the expression was induced in SGCAA medium (20 g/liter galactose, 20 g/liter raffinose, 1 g/liter dextrose, 6.7 g/liter Difco yeast nitrogen base w/o amino acid, 5 g/liter Bacto casamino acids, 5.4 g Na_2_HPO_4_, and 8.56 g/liter NaH_2_PO_4_·H_2_O) according to published protocols [39]. For shFcRn binding, 5×10^5^ yeast cells were harvested, washed by PBSA (PBS+0.1% bovine serum albumin), pH 6.0 and re-suspended in 50 µl PBSA (pH 6.0) containing 100 nM biotin-conjugated shFcRn. The samples were kept on ice for 2 hours, then the cells were washed by PBSA (pH 6.0) again and still re-suspended in 50 µl PBSA (pH 6.0) with 1 µl PE-streptavidin (Invitrogen, CA). After 45-min incubation on ice, the cells were washed by PBSA (pH 6.0) and re-suspend in 0.5 ml PBSA (pH 6.0) for flow cytometry measurement. Yeast-expressed Fc [22] was used as control. The mouse anti-human CH2 mAb (Abd sterotec, NC) described in our previous work [22] and Alexa Fluor 488-conjuated goat anti-mouse IgG (Invitrogen, CA) were used for testing the expression of m2a1 the yeast. Same samples prepared at pH 7.4 were used as controls.

### Binding of m2a1 to shFcRn Measured by ELISA

To confirm the result from flow cytometry, we also used ELISA to test the binding of m2a1 to shFcRn at pH 6.0 and pH 7.4. Fc expressed from *E. coli* was used as positive control. Purified shFcRn was diluted in the PBS buffer in concentration of 2 µg/ml and coated to the 96-well plate at 4°C overnight. Serially diluted m2a1 and Fc from 0 to 1000 nM in PBSA, pH 6.0 and pH 7.4, respectively, was added to each wells. HRP-conjugated goat anti-human Fc antibody (Sigma-Aldrich, MO) was used as secondary antibody. ABTS described above was also used for color development.

### Formation of shFcRn/m2a1/sp62 Complex

Purified shFcRn was diluted in the PBS buffer in concentration of 2 µg/ml and coated to the 96-well plate at 4°C overnight. m2a1 (500 nM) and Fc (500 nM) were added into each wells for 2-hour incubation at 37°C. Then pre-mixed biotin-sp62 peptide and Streptavidin-Peroxidase Polymer as described above in pH 6.0 and pH 7.4, respectively, were added. After 2-hour incubation at 37°C, color was developed after adding ABTS.

### Molecular Modeling and Docking of hFcRn/m2a1/sp62 Complex

The initial coordinates of m2a1 were constructed via homology modeling (http://swissmodel.expasy.org) [40]–[42] based on a 1.65 Å Fc structure (pdb entry: 1L6X) [43]. A water box was then added using VMD 1.8.6 program [44] and water molecules were retained in the molecular dynamics simulation. The m2a1 was simulated with CHARMM force field using NAMD program [45]. The structure was first minimized for 5000 steps with conjugate gradient method, and equilibrated for 10 ps with the time step of 1 fs, then were further minimized for 90,000 steps for analysis with VMD program. The coordinates of gp41 MPER-derived peptide sp62 was obtained from a complex structure of sp62 with an IgG Fab fragement (pdb entry: 3MNW) [46]. Docking simulation of sp62 with energy-minimized m2a1 was performed using the Rosetta-Dock program. At last, the docked sp62-m2a1 and human FcRn (pdb entry: 3M17 [47]) were superimposed onto the complex structure of rat FcRn-Fc (pdb entry: 1I1A) [48], and were welled matched with rat IgG2a Fc CH2 domain and rat FcRn, respectively.

## Results

### Generation of an m01s-based Library

To construct diversified m01s-based libraries containing human sequences we developed a novel method for precise grafting of H3s from our VH-based libraries containing human H3s. Because of the H3 variability, precise replacement of the m01s loop FG (which is most similar to H3) with H3s is not possible by a single step PCR. By analyzing the FG flanking DNA sequences we designed a complex multi-step PCR method (Fig. 1A and Table 1) which allowed preserving the exact sequences of the FG flanking fragments. This method was initially tested with a known VH-based engineered domain (eAd), m36 [24]. According to the sequence alignment of CH2 and m36 based on the conserved disulfide bond (Fig. 1B), we found that loop BC in CH2 could match H1 while loop FG in CH2 could match H3. However, CH2 loop DE did not match the corresponding partner H2 in m36. m01s was still monomeric after grafting of the H3 (Fig. 1C). These results indicate that H3 could be grafted onto m01s loop FG for introduction of both sequence and length diversities.

We also analyzed the frequency of occurrence of specific amino acids in germline H1s (Fig. 2 and Table 2). The dominant length of the H1 was eight residues. Therefore, we deleted the “D” on the tip of loop BC and rationally mutated the other eight residues according to the analysis of germline H1s. We also did limited mutagenesis of loop DE according to the property of each residue (Table 3). By combining grafting and mutagenesis, we generated a 10^9^ phage-displayed library. We randomly selected 25 clones for sequencing and found that 19 clones could be expressed correctly (Fig. 3). They had different sequences in all the three loops and various lengths from 9 residues to 23 residues in loop FG, which is indicative of the high quality of this library.

### Selection and Characterization of Antigen Binding Properties of m2a1

After five rounds of panning against biotin-sp62, two dominant clones m2a1 (3 copies) and m2a2 (4 copies) were indentified from 10 clones, which were solubly expressed (Fig. 4). Only m2a1 was chosen for further characterization because m2a2 was difficult to be purified and exhibited tendency for aggregation. m2a1 (∼15 kD) was monomeric as measured by SEC (Fig. 5). m2a1 bound to the antigen biotin-sp62 as measured by ELISA with EC50 = 51 nM (Fig. 6A, left) while m66.6 bound to sp62 more strongly (EC50 = 4 nM) (Fig. 6A, right). No binding of m2a1 to biotin-scrambled sp62 peptide was observed indicating that the binding of m2a1 to sp62 was specific. m2a1 neutralized a panel of HIV-1 isolates in a cell line-based pseudovirus assay although with relatively low potency (Fig. 6B). In an attempt to elucidate the mode of m2a1 binding to sp62 we generated three mutants in which each of the loops was mutated back to the original sequence of the scaffold. When any loop in m2a1 was reverted back to its corresponding loop in m01s, the binding to sp62 was aborted as measured by ELISA (Fig. 7) indicating all three loops in m2a1 were involved in the binding.

### shFcRn Binds to m2a1 Binds and does not Compete with sp62

Recently, we showed that m01s expressed on yeast surface binds to shFcRn in a pH-dependent manner [22]. Here, the same method was used to test the binding of m2a1 to shFcRn at pH 6.0 and pH 7.4. The expression level of m2a1 on yeast surface was checked by using a mouse anti-human CH2 mAb. More than 60% of the yeast cells displayed m2a1 on their surface (first figure in fig. 8A, left peak: tranfected yeast cells without m2a1 expression, right peak: transfected yeast cells with m2a1 expression). The shift of the fluorescence intensity as measured by flow cytometry when pH was changed from 7.4 to pH 6.0 indicated the binding of m2a1 to shFcRn was pH-dependent. There was no shift if only PE-streptavidin was added indicating that the pH-dependent binding of m2a1 to shFcRn was specific. Fc expressed on yeast surface was used as positive control. ELISA was also used to test the binding of purified m2a1 to shFcRn (Fig. 8B). Binding of m2a1 to shFcRn was observed at pH 6.0 but not at pH 7.4, which is consistent with the results from the flow cytometry. Bacterially expressed Fc was used as a positive control. The binding of Fc to shFcRn at pH 6.0 was stronger than that of m2a1s to shFcRn. Very weak binding of Fc to shFcRn at pH 7.4 was also found.

To find whether the binding sites for sp62 and FcRn overlap, we measured binding of m2a1 to sp62 in the presence of shFcRn. Binding of m2a1 to sp62 was not affected by shFcRn at pH 6.0 as measured by ELISA (Fig. 9), which indicated that sp62 and FcRn were noncompetitively interacting with m2a1. According to the crystal structure of a rat FcRn/rat Fc complex and results from identification of amino acids on human CH2 domain that are critical for binding to human FcRn by site-directed mutagenesis [48], [49], the FcRn binding sites on CH2 mainly locate at the helix regions (see below) that are on the opposite side of the three loops. Therefore, the antigen sp62 did not interfere with the binding of m2a1 to shFcRn.

### Modeling of the FcRn/m2a1/sp62 Complex

Based on the available rat crystal structure and our experimental results, we developed a model of the shFcRn/m2a1/sp62 complex by molecular modeling (Fig. 10). According to this model m2a1 binds to sp62 mostly by the grafted H3– a mode of binding similar to the binding of 2F5 to sp62 [50]. It could also contact FcRn by residues in helix-1, which is similar to the binding of Fc to FcRn [48]. Although we found that all three loops in m2a1 were involved in binding, only loop FG was predicted to directly bind to sp62 in this model. One could speculate that loop BC and loop DE may contribute to the binding through conformational changes of the structure of m2a1 without contacting sp62 directly. This model provides a possible structural mechanism explaining the simultaneous binding of m2a1 to two molecules.

## Discussion

The small size of nAbs may lead to relatively good penetration into tissues and the ability to bind into cavities or active sites of protein targets which may not be accessible to full size antibodies [7]. This could be particularly important for the development of therapeutics against rapidly mutating viruses, e.g., HIV-1. Because these viruses have evolved in humans to escape naturally occurring antibodies of large size, some of their surface regions which are critical for the viral life cycle may be vulnerable for targeting by molecules of smaller size. In our previous study, we showed that the stability of CH2 could be dramatically increased. Therefore, some nAbs may be more stable than full size antibodies in the circulation. They could be taken orally or delivered via the pulmonary route or even penetrate the blood-brain barrier, and retain activity even after being subjected to harsh conditions, such as freeze-drying or heat denaturation. In addition, nAbs could be monomeric, of high solubility and relatively low aggregation propensity or can be engineered to reduce aggregation. The nAbs described here are human molecules which decreases the likelihood of undesirable immune responses. Importantly, their half-life in the circulation can be relatively easily adjusted from minutes or hours to weeks. Therefore, nAbs are promising representatives of a novel type of candidate therapeutics [51]. Our previous data showed that m01s, a modified version of CH2, not only exhibits improved biophysical and biochemical properties as a scaffold but also binds to FcRn at pH 6.0 and not at pH 7.4 [22]. Recently, we measured the half-lives of m01s in three different animal models: normal B6 mice, human FcRn transgenic mice and cynomolgus macaques [52]. The results showed that m01s has relatively long (about 10 hours) half-life compared to other scaffolds of similar size possibly due to its binding to FcRn.

We have previously identified a binder (m1a1) based on wtCH2 scaffold against HIV-1 gp120 [17]. However, we also found that binding of CH2 to FcRn was very weak and the serum half-life of CH2 *in vivo* was shorter than that of m01s [22], [52]. We therefore used a novel strategy based on multi-step PCR to construct a library based on the m01s as a scaffold. The multi-step PCR resulted in the precise grafting of the H3 onto loop FG in m01s. After panning this library against an HIV-1 MPER peptide sp62, we obtained two dominant clones m2a1 and m2a2. m2a2 exhibited relatively high propensity for aggregation and was not further characterized. We found that m2a1 interacts noncompetitively with an HIV-1 neutralizing epitope and FcRn, which shows that eAds could mimic both antigen-binding and effector/stability functions of full-size mAbs. This could have important implications for the stability and related pharmacokinetics *in vivo*. A major drawback for some applications of small-size binders is their short half-life (typically on the order of minutes [10], [53]) in serum partially due to lack of FcRn binding. Similarly to the scaffold m01s, m2a1 also binds to FcRn in a pH-dependent manner. It has been previously shown that the pH-dependent binding to FcRn is important for the half-life *in vivo*; if the binding of FcRn at pH6.0 and pH7.4 increases simultaneously, the pharmacokinetics is not significantly improved [54]. Since the m01s half-life is about 10-hour [52], it is quite probably that the half-life of m2a1 is similar and relatively long for proteins of such small size.

Our results show for the first time that single eAds could be generated that bind simultaneously to both antigen and FcRn, and provide a proof of concept that such eAds could be selected from a semi-synthetic CH2-based library. However, the propensity for aggregation of CH2-based eAds appears high and the other clone which was selected aggregated. This could be due to the library design based on isolated CH2 which although stabilized to m01s is not further modified to increase resistance to aggregation. In progress are experiments to develop better libraries containing potential binders with higher resistance to aggregation. Further studies are also needed to test in animal models and in humans the utility of this new concept – eAds mimicking certain full-size antibody functions (nanoantibodies) – for the development of novel type of candidate therapeutics and diagnostics.
